# Comparative Analysis of Urine Fractions for Optimal Bladder Cancer Detection Using DNA Methylation Markers

**DOI:** 10.3390/cancers12040859

**Published:** 2020-04-02

**Authors:** Anouk E. Hentschel, Jakko A. Nieuwenhuijzen, Judith Bosschieter, Annina P. van Splunter, Birgit I. Lissenberg-Witte, J. Patrick van der Voorn, Loes I. Segerink, R. Jeroen A. van Moorselaar, Renske D.M. Steenbergen

**Affiliations:** 1 Urology, Cancer Center Amsterdam, Amsterdam UMC, Vrije Universiteit Amsterdam, de Boelelaan 1118, 1182 DB Amsterdam, The Netherlands; a.hentschel@amsterdamumc.nl (A.E.H.); j.nieuwenhuijzen@amsterdamumc.nl (J.A.N.); j.bosschieter@amsterdamumc.nl (J.B.); rja.vanmoorselaar@amsterdamumc.nl (R.J.A.v.M.); 2 Pathology, Cancer Center Amsterdam, Amsterdam UMC, Vrije Universiteit Amsterdam, de Boelelaan 1118, 1182 DB Amsterdam, The Netherlands; a.vansplunter@amsterdamumc.nl (A.P.v.S.); jp.vandervoorn@amsterdamumc.nl (J.P.v.d.V.); 3 Epidemiology & Biostatistics, Amsterdam Public Health, Amsterdam UMC, Vrije Universiteit Amsterdam, de Boelelaan 1117, 1081 HV Amsterdam, The Netherlands; b.lissenberg@amsterdamumc.nl; 4 BIOS Lab on a Chip Group, MESA + Institute for Nanotechnology, Technical Medical Centre, University of Twente, Hallenweg 15, 7522 NH Enschede, The Netherlands; l.i.segerink@utwente.nl

**Keywords:** biomarkers, methylation, molecular diagnostics, urinary bladder neoplasms, urine analysis

## Abstract

DNA methylation analysis of full void urine and urine pellet seems promising for bladder cancer (BC) detection and surveillance. Urinary cell-free DNA from urine supernatant is now gaining interest for other molecular tests in BC. This study aims to evaluate which urine fraction is preferred for BC diagnosis using methylation markers: full void urine, urine pellet or supernatant. Methylation levels of nine markers were determined in the three urine fractions and correlated with their respective tumor tissues in BC patients and compared to controls. For all markers and marker panel *GHSR/MAL*, diagnostic performance was determined by calculating the area under the curve (AUC) of the respective receiver operating characteristic curves. For most of the markers, there was a significant correlation between the methylation levels in each of the urine fractions and the matched tumor tissues. Urine pellet was the most representative fraction. Generally, AUCs for BC diagnosis were comparable among the fractions. The highest AUC was obtained for *GHSR/MAL* in urine pellet: AUC 0.87 (95% confidence interval: 0.73–1.00), corresponding to a sensitivity of 78.6% and a specificity of 91.7%. Our results demonstrate that cellular and cell-free DNA in urine can be used for BC diagnosis by urinary methylation analysis. Based on our comparative analysis and for practical reasons, we recommend the use of urine pellet.

## 1. Introduction

Hematuria is often the first sign of disease in bladder cancer (BC) patients. At referral, an endoscopic inspection of the bladder wall through cystoscopy and imaging of the upper urinary tract is performed. In case BC is diagnosed, regular follow-up cystoscopies are required for multiple years, since disease often recurs or progresses [1]. In the future, biomarker analysis in urine, such as DNA hypermethylation, may be an alternative and effective approach for BC detection and surveillance. DNA methylation refers to the addition of a methyl group to cytosine residues in CpG dinucleotides. Hypermethylation of tumor suppressor gene promoter regions results in gene silencing and is known to be associated with the development of BC [2]. Many potential urinary methylation markers for BC detection have been investigated, with great variety in diagnostic performance [3,4,5].

In our previous study, we showed that full void urine, comprising both cellular and cell-free DNA, can be used for DNA methylation analysis in BC patients. We demonstrated an optimal performance of the biomarker panel *GHSR/MAL*, with a sensitivity of 92% (95% confidence interval [CI]: 86–99) at a specificity of 85% (95% CI: 76–94), for BC diagnosis in full void urine [6]. The ability to detect DNA hypermethylation in urine is most likely a consequence of the direct shedding of bladder tumor cells into the urine [7]. Based on this assumption, most studies performed to date collected cells by centrifugation of the urine and isolated DNA from cell pellets [3]. Two studies revealed that cell-free DNA from urine supernatant was more sensitive for the detection of tumor-derived copy number alterations, loss of heterozygosity and somatic mutations than cellular DNA from urine pellet [8,9]. As cell-free DNA is suggested to originate from apoptosis and necrosis of cancer cells and may therefore be more enriched for bladder tumor DNA, this might also be an interesting alternative for DNA methylation analysis [8,9,10].

In this technical report, we aim to compare full void urine, urine pellet or supernatant for urine-based BC diagnosis using DNA methylation markers. To our knowledge, such a comparative study on urine fractions for DNA methylation analysis in BC patients is not yet available. For this purpose, we selected nine promising methylation markers from our previous study. These encompass seven protein-coding genes (*FAM19A4, GHSR, MAL, PHACTR3, PRDM14, SST* and *ZIC1*)—of which, the CpG islands are methylated in bladder cancer—as well as two miRNAs (*miR-129* and *miR-935*) with promoter regions that are also known to be methylated in bladder cancer [6]. We examined the correlation between the methylation levels of the nine markers between each of the three urine fractions and the matched bladder tumor tissues in 14 BC patients. Subsequently, we included a control group of 12 benign hematuria cases to compare the diagnostic potential of methylation analysis in the three urine fractions.

We demonstrate that all three urine fractions are suitable for urinary methylation analysis, thereby providing first evidence for BC detection in urine supernatant by methylation markers. Nonetheless, we recommend urine pellet over the other two fractions based on the results of our comparative analysis and practical considerations. 

## 2. Results

Characteristics of all included patients are summarized in Table 1. Most BC patients (11/14) were male. Median age was 70.0 years (Interquartile range [IQR]: 62.5–79.3) in BC patients and 58.5 years (IQR: 49.3–70.8) in controls (*p =* 0.033). Stage and grade of disease varied among the BC patients. Non-muscle-invasive disease was present in 12 and muscle-invasive disease in 2 patients. Low- and high-grade disease were diagnosed in eight and six BC patients, respectively.

Urine fractions were obtained from one urine sample per patient. Invalid test results led to the exclusion of four urine fractions, after which 74 urine fractions were left for methylation analysis.

### 2.1. Correlation Between the Urine Fractions and Matched Tumor Tissues

For all BC patients, methylation levels of the nine markers were determined in the three urine fractions and correlated with their respective tumor tissues (Table 2). In full void, methylation levels of *GHSR, miR-129, PRDM14, SST* and *ZIC1* were significantly correlated to the matched tumor tissues. In urine pellet, methylation levels of all markers, except *miR-935*, were significantly correlated with the matched tumor tissues. For urine supernatant, there was a significant correlation between methylation levels of *MAL, PHACTR3, PRDM14, SST* and *ZIC1* and the matched tumor tissues.

Highest correlations (>0.70) were found for *GHSR* (urine pellet), *MAL* (urine pellet and supernatant), *miR-129* (full void), *PRDM14* (full void and urine pellet) and *SST* (full void).

### 2.2. Discriminative Capability of the Urine Fractions

**Figure 1:**
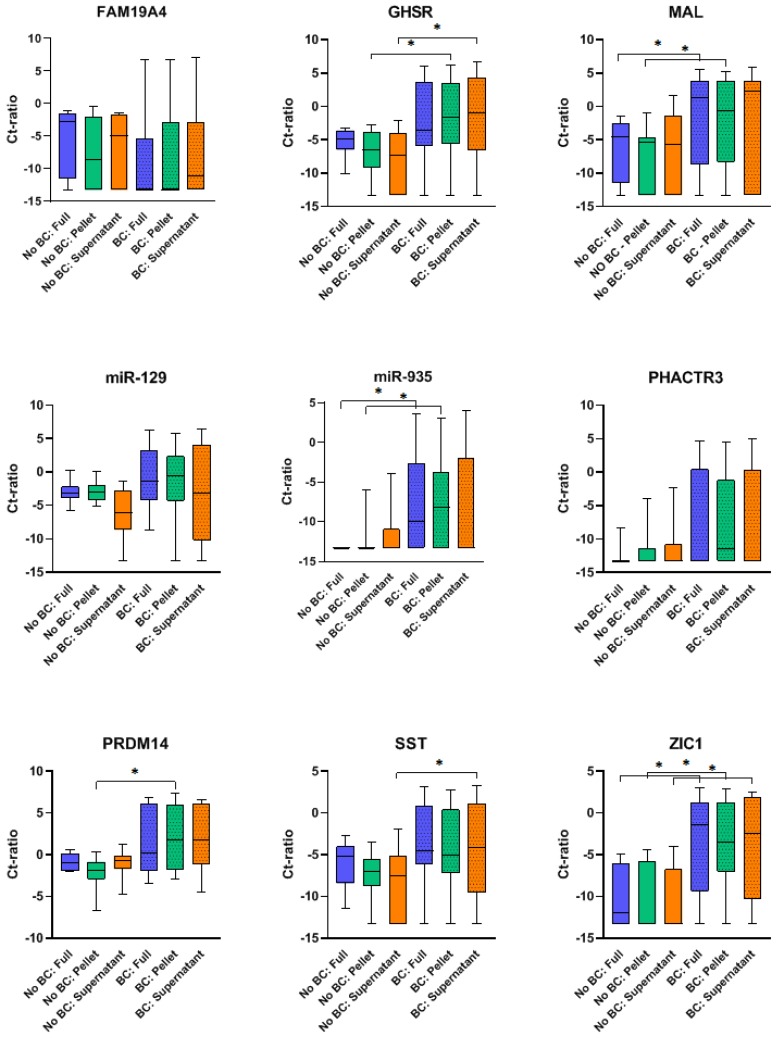
Box plots of methylation levels for bladder cancer (BC) patients and benign hematuria controls in each urine fraction (full void, pellet and supernatant) per methylation marker. Significant outcomes (*p <* 0.05) of the Mann–Whitney U test are displayed as *.

Both BC patients and hematuria controls were included to determine the discriminative capability of the methylation markers in the different urine fractions for BC diagnosis. In Figure 1, methylation levels of all nine methylation markers in each urine fraction are displayed for BC patients and hematuria controls. In full void, methylation levels of *MAL*, *miR-935* and *ZIC1* were significantly higher in BC patients than in hematuria controls. For urine pellet, *GHSR, MAL, miR-935, PRDM14* and *ZIC1* had significantly higher methylation levels in BC patients. In urine supernatant, methylation levels of *GHSR, SST* and *ZIC1* significantly differed between BC patients and hematuria controls.

### 2.3. Diagnostic Potential of the Urine Fractions

For each of the urine fractions, the area under the curve (AUC) for BC diagnosis was calculated for the nine methylation markers and marker panel *GHSR/MAL* (Table 3). For most markers, comparable AUC values were obtained for the three urine fractions. Of the single methylation markers, *ZIC1* performed best, with AUC values of 0.78 (95% CI: 0.60–0.96) to 0.81 (95% CI: 0.64–0.98) among urine fractions. The highest AUC was obtained for marker panel *GHSR/MAL*, with an AUC of 0.87 (95% CI: 0.73–1.00) in urine pellet. Sensitivity and specificity results are reported in Table S1, in which the values are based on Youden’s J index and do not necessarily reflect an optimal diagnostic setting.

Next, we determined the relative sensitivity and specificity of urine pellet and supernatant over full void, using the optimal thresholds from our previous study for full void. For *miR-935*, sensitivity was significantly higher in urine pellet than in full void (Table 4). A subset of the other individual markers (*GHSR, MAL, miR-129, PHACTR3, PRDM14* and *SST*) had a higher, though non-significant, relative sensitivity for urine pellet and/or supernatant over full void (Table 4). For the *GHSR/MAL* combination, sensitivity was also higher in urine pellet than in full void, i.e., a relative sensitivity of 1.43 (95% CI: 0.95–2.14), at a relative specificity of 0.92 (95% CI: 0.77–1.09).

## 3. Discussion

In this paper, we compared three urine fractions for BC detection through DNA methylation analysis. In BC patients, we found a significant correlation between the methylation levels in each of the urine fractions and the matched tumor tissues for most of our markers. We confirmed the results of our previous study, in which we used DNA methylation analysis of full void urine for BC diagnosis [6]. Present data indicate that urine pellet and supernatant can be used for this purpose as well. Our optimal marker panel *GHSR/MAL*, performed particularly well in urine pellet, with an AUC of 0.87 (95% CI, 0.73–1.00) for BC diagnosis, corresponding to a sensitivity of 78.6% (95% CI, 49.2–95.3) and a specificity of 91.7% (95% CI, 61.5–99.8).

The detection of DNA hypermethylation in urine pellet from BC patients has been studied extensively as a fluid biomarker. This is generally considered the most convenient specimen for BC diagnosis, since it is expected that bladder tumor cells that are directly shed into the urine descend to the pellet upon centrifugation [3,10,11]. However, the presence of ‘background’ DNA from normal cells makes the sensitive detection of hypermethylated DNA in urine pellet challenging, particularly in low-grade/-stage disease [11,12,13]. As cell-free DNA from urine supernatant is assumed to be enriched for tumor DNA, this appeared an interesting alternative for urine-based methylation analysis for BC diagnosis [8,9,10,11]. We have shown that urinary cell-free DNA can indeed be used for BC detection by DNA methylation analysis. 

So far, little is known about the origin of hypermethylated cell-free DNA in urine of BC patients. It has been suggested to result from apoptosis and/or necrosis of bladder tumor cells, but it may also enter the urinary tract through the blood circulation and kidney barrier. Either mechanism could also depend on the stage and/or grade of the bladder tumor present [10,11]. Theoretically, less cell-free DNA is to be expected in urine of low-grade tumors which generally display less apoptosis and necrosis than high-grade tumors.

In general, we observed only small differences in urinary DNA methylation analysis between the fractions. Interestingly, we found that the strength of the correlation between the urinary methylation levels of a marker and the matched tumor tissues did not always correspond with the diagnostic potential of the marker. For example, the strongest correlation between urinary methylation levels and matched tumor tissues was found for *MAL* in urine supernatant (0.83, *p <* 0.001). However, BC patients could not be discriminated from controls based on their *MAL* methylation levels in urine supernatant and the AUC was rather low: 0.69 (95% CI: 0.46–0.91). Vice versa was observed for *MAL* in urine pellet. In our previous study, we examined both bladder tumor tissues and benign bladder biopsies and found that methylation of both *MAL* and *GHSR* was higher in bladder tumor tissues, which underlines the diagnostic utility of these markers [6]. As the diagnostic potential of a marker also depends on the urinary methylation levels of controls, the presence of normal ‘background’ DNA in benign hematuria patients might be of influence. Varying amounts of ‘background’ DNA in the urine fractions of benign hematuria controls may explain the differences between urine fractions. This is illustrated by Cheng et al. (2019), who showed that blood cells partly determine the methylation profile in urinary cell-free DNA of benign hematuria controls [14].

Based on the results of our comparative analysis, we recommend urine pellet for urine-based BC diagnosis using DNA methylation markers. When practical reasons are considered, urine pellet is also preferred, as it is cheaper and faster to process. However, interestingly, the favorable performance of urine supernatant was also noted. To our knowledge, this is first study that reports on DNA methylation markers in cell-free DNA for urine-based BC detection. Considering ongoing technological developments, such as the use of nanotechnology, we also envision the utility of DNA methylation analysis in urinary cell-free DNA in future BC patients. Particularly, since recent publications already showed that BC can be detected from cell-free DNA in urine with other techniques, including the analysis of DNA fragment lengths and the detection of molecular alterations, such as loss of heterozygosity [8,9,14]. A combination of urinary DNA methylation analysis and other existing techniques, e.g., DNA mutation analysis, might further improve the diagnostic potential of a urine-based BC test [15,16,17].

This study has a few limitations. Firstly, the sample size was too small to draw strong conclusions about the diagnostic accuracy of the methylation markers. Nonetheless, numbers were sufficient for a technical comparison, including the analysis of 78 urine fractions and 14 tumor tissues of BC patients for 9 methylation markers. Secondly, benign hematuria controls were younger (*p =* 0.033) and more often female (*p =* 0.054) than the BC patients. This might have introduced a bias for the diagnostic potential, since our previous study showed lower sensitivities in females for all nine methylation markers, at similar specificities [6]. However, in a systematic review on urinary methylation markers, there was no evidence for a difference in diagnostic accuracy for gender [3]. Furthermore, tumor characteristics of BC patients were heterogeneous, which is in accordance with clinical practice, but this might have influenced the diagnostic potential of our markers. We believe that this did not impair our technical comparison. Strengths of this study are the simultaneous collection of urine fractions and the matched comparison with tumor tissues in BC patients, the inclusion of hematuria controls and the usage of a robust quantitative methylation specific PCR (qMSP) assay [6,18,19,20,21,22].

## 4. Materials and Methods

### 4.1. Patients

We included 14 urothelial BC patients from Amsterdam UMC, location VUmc. Urine and matched tumor tissues were obtained from all BC patients. The presence of non-muscle-invasive or muscle-invasive disease was histologically confirmed. The World Health Organization (WHO) 1973 (Grade 1–3) and 2004 (Low- or High-Grade) grading systems were used for histological grading of the tissues [23,24]. The control group consisted of 12 benign hematuria controls without malignancy at cystoscopy and upper urinary tract imaging, and without a history of previous malignancy. The study was conducted in accordance with the Declaration of Helsinki, and the protocol was approved by the medical ethical committee of Amsterdam UMC, location VUmc (2018.355). All patients gave informed written consent for study participation.

### 4.2. Preparation of Urine and Tissue Specimens

Urine samples were prospectively collected prior to cystoscopy or surgery. Urine samples were preserved by adding ethylenediaminetetraacetic acid in a final concentration of 40 mM [25]. From each participant, we stored 30–40 mL full void urine at −20 °C in the first 24 h after collection and we used 15 mL for centrifugation. Urine samples were centrifuged at room temperature at 800× *g* for 10 min to obtain a pellet and supernatant and both were stored at −20 °C.

Tumor tissues were fixed in formalin and embedded in paraffin, as part of standard clinical practice. An experienced uro-pathologist (Dr. J.P. van der Voorn) reviewed histological grade and stage of the 4 µm cut and hematoxylin and eosin (H&E) stained sections. The archived whole tumor tissues were serially sectioned (10 µm) using a microtome according to the sandwich cutting technique. Presence of tumor tissue was histologically confirmed in the first and last obtained section (3 µm) after H&E staining. The other sections were stored in sterile polymerase chain reaction (PCR) tubes in preparation for DNA isolation [26].

### 4.3. DNA Isolation and Bisulfite Conversion

DNA from full void and urine supernatant was isolated with the QuickDNA™ Urine Kit (Zymo Research, Orange, CA, USA). For DNA isolation from urine pellet, the QIAamp DNA Mini Kit (Qiagen GmbH, Hilden, Germany) was used. For DNA isolation from histological tissue, the QIAamp DNA FFPE Tissue Kit (Qiagen GmbH, Hilden, Germany) was used. Next, bisulfite conversion was performed using the EZ DNA Methylation™ Kit (Zymo Research, Orange, CA, USA). The manufacturer’s protocols were followed for DNA isolation and bisulfite conversion. 

### 4.4. Quantitative Methylation Specific PCR

DNA methylation analysis was performed after bisulfite conversion. Multiplex qMSP was performed for the genes *FAM19A4, GHSR, MAL, miR-129, miR-935, PHACTR3, PRDM14, SST* and *ZIC1* as described previously [6,18,19,20,21,22]. For the multiplex qMSP, we used 50 ng of bisulfite-converted DNA, 200–300 nmol/L of the primers and fluorescent dye-labeled probe, on the ABI 7500 Fast Real-Time PCR System (Applied Biosystems, CA, USA) and the ViiA™ 7 Real-Time PCR System (Applied Biosystems, CA, USA).

We used positive controls (bisulfite-converted DNA of BC cell lines *TCC-SUP* and *J82*) and a negative control (H_2_O). The comparative Ct method (2^−∆CT^ × 100) was used to normalize the methylation values of the targeted genes to the reference gene *ACTB*, resulting in Ct ratios of the targeted genes [27]. Four urinary fractions with an *ACTB* Ct >32 were considered invalid and were therefore excluded from data analysis.

### 4.5. Statistical Analyses

Frequencies and percentages were used as descriptive statistics for categorical data, and median, first and third quartiles for continuous data. Between BC patients and controls, categorical data were compared with the Chi-square test and medians of continuous data with the Mann–Whitney U test. Ct ratios between the targeted genes and reference gene *ACTB* were first log2 transformed and were then analyzed. Correlation between Ct ratios was determined through Spearman’s correlation. For all single markers and the marker panel *GHSR/MAL*, a receiver operating characteristic (ROC) curve was made and the AUC was calculated. The ROC of *GHSR/MAL* was obtained by scoring the number of positive markers in the panel (0, 1 or 2) per patient. 

Sensitivity and specificity for BC diagnosis of urine pellet and supernatant were compared with full void by calculating relative sensitivity and specificity as well as the 95% CI. For urine pellet and supernatant, new cut-offs for the Ct ratios of the targeted genes were calculated with Youden’s J index; for full void, cut-offs for the Ct ratios of the targeted genes were in accordance with our previous study (Table S2) [28,29]. The sensitivity and specificity of *GHSR/MAL* was determined according to ‘believe the positive’: *GHSR/MAL* was positive if either *GHSR, MAL* or both markers were positive [30]. Sensitivity and specificity of urine pellet and supernatant were considered to be statistically significantly higher than that of full void when the lower bound of the 95% CI was above 1, statistically significantly lower when the upper bound of the 95% CI was below 1 and not statistically significantly different when the 95% CI contained the value 1. Statistical analyses were performed with SPSS Software (SPSS 22.0, IBM Corp., NY, USA) and R version 3.5.3 (R Foundation for Statistical Computing, Vienna, Austria). Tests were two sided and statistical significance was assumed if *p <* 0.05.

## 5. Conclusions

This comparative study demonstrates the suitability of all three urine fractions for urinary methylation analysis based on the minor differences in diagnostic performance observed between the urine fractions. Hereby, we show for the first time that urine supernatant is also suitable. Nonetheless, we recommend urine pellet over the other two fractions for three reasons. Firstly, urine pellet represents the matched tumor tissues best, as eight out of nine markers show a significant correlation. Secondly, despite the similar diagnostic performance of all three fractions, urine pellet discriminates best between BC patients and controls for our optimal marker panel *GHSR/MAL*. Thirdly, urine pellet is the cheapest and fastest to process.

## 6. Patents

Judith Bosschieter, Jakko A. Nieuwenhuijzen, Loes I. Segerink and Renske D.M. Steenbergen are inventors on patents related to the work.

## Figures and Tables

**Table 1:** Baseline characteristics of all included patients.

Characteristic	Bladder Cancer (*n =* 14)	Benign Hematuria (*n =* 12)	*p*-Value
Sex, *n* (%)			*0.054*
Male	11 (78.6)	5 (41.3)	
Female	3 (21.4)	7 (58.3)	
Age, yr, *median (IQR)*	70.0 (62.5–79.3)	58.5 (49.3–70.8)	*0.033*
WHO 1973 grade, *n (%)*			-
Grade 1	2 (14.3)	-	
Grade 2	7 (50.0)	-	
Grade 3	5 (35.7)	-	
WHO 2004 grade, *n (%)*			-
Low-grade	8 (57.1)	-	
High-grade	6 (42.9)	-	
Stage, *n (%)*			-
Ta	9 (64.3)	-	
Tis	1 (7.1)	-	
T1	2 (14.3)	-	
T3b	2 (14.3)	-	
Primary/recurrence, *n (%)*			-
Primary	8 (57.1)	-	
Recurrence	6 (42.9)	-	
Number of tumors, *n* (%)			-
Solitary	8 (57.1)	-	
Multiple	6 (42.9)	-	
Tumor size, *n* (%)			-
<3 cm	11 (78.6)	-	
≥3 cm	3 (21.4)	-	
CIS, *n* (%)			-
No	12 (85.7)	-	
Yes	2 (14.3)	-	

Abbreviations: yr = year; IQR = interquartile range; WHO = World Health Organization; CIS = carcinoma in situ.

**Table 2:** Correlation between the methylation levels in the urine fractions and matched tumor tissues of the bladder cancer patients (*n =* 14). Spearman’s correlation was used to calculate the correlation coefficients and corresponding *p*-values.

	Spearman’s Correlation		
Markers	Full Void vs. Tissue	Pellet vs. Tissue	Supernatant vs. Tissue
	Correlation Coefficient (*p*-Value)	Correlation Coefficient (*p*-Value)	Correlation Coefficient (*p*-Value)
***FAM19A4***	0.30 (*0.32*)	**0.57 (*0.035*)**	0.35 (*0.23*)
***GHSR***	**0.60 (*0.031*)**	**0.77 (*0.001*)**	0.55 (*0.050*)
***MAL***	0.55 (*0.050*)	**0.74 (*0.002*)**	**0.83 (*0.001*)**
***miR-129***	**0.78 (*0.002*)**	**0.58 (*0.029*)**	0.53 (*0.062*)
***miR-935***	0.34 (*0.26*)	0.47 (*0.093*)	0.34 (*0.26*)
***PHACTR3***	0.47 (*0.10*)	**0.58 (*0.031*)**	**0.55 (*0.044*)**
***PRDM14***	**0.75 * (0.003*)**	**0.74 (*0.002*)**	**0.61 (*0.021*)**
***SST***	**0.76 (*0.003*)**	**0.68 (*0.008*)**	**0.60 (*0.029*)**
***ZIC1***	**0.57 (*0.044*)**	**0.69 (*0.006*)**	**0.62 (*0.023*)**

Values in bold represent statistically significant correlations (i.e., *p* < 0.05).

**Table 3:** Area under the curve values for the nine methylation markers and marker panel *GHSR/MAL*, in full void, urine pellet and supernatant.

Markers	Full Void	Pellet	Supernatant
		AUC (95% CI)	
***FAM19A4***	0.31 (0.09–0.54)	0.41 (0.18–0.63)	0.43 (0.20–0.65)
***GHSR***	0.69 (0.47–0.90)	0.78 (0.60–0.97)	0.78 (0.58–0.97)
***MAL***	0.75 (0.55–0.96)	0.74 (0.54–0.94)	0.69 (0.46–0.91)
***miR-129***	0.67 (0.45–0.90)	0.63 (0.40–0.86)	0.64 (0.42–0.87)
***miR-935***	0.81 (0.63–0.99)	0.75 (0.56–0.94)	0.64 (0.42–0.86)
***PHACTR3***	0.66 (0.45–0.87)	0.66 (0.45–0.87)	0.63 (0.42–0.85)
***PRDM14***	0.62 (0.38–0.85)	0.79 (0.61–0.97)	0.73 (0.53–0.93)
***SST***	0.68 (0.46–0.90)	0.70 (0.50–0.91)	0.73 (0.53–0.94)
***ZIC1***	0.81 (0.64–0.98)	0.78 (0.60–0.96)	0.80 (0.62–0.98)
**Panel *GHSR/MAL***	0.77 (0.58–0.96)	0.87 (0.73–1.00)	0.83 (0.66–1.00)

Abbreviations: AUC, area under the curve; CI, confidence interval.

**Table 4:** Relative sensitivity and specificity of urine pellet and supernatant for bladder cancer diagnosis, as compared to full void. New cut-offs were determined for urine pellet and supernatant; optimal cut-offs from our previous study were used for full void [6].

Markers	Pellet		Supernatant	
	Relative Sens: Pellet vs. Full Void (95% CI)	Relative Spec: Pellet vs. Full Void (95% CI)	Relative Sens: Supernatant vs. Full Void (95% CI)	Relative Spec: Supernatant vs. Full Void (95% CI)
***FAM19A4***	1.00 *	1.00 *	1.00 *	1.00 *
***GHSR***	1.60 (0.94–2.74)	1.00 *	1.40 (0.88–2.24)	1.00 *
***MAL***	1.50 (0.95–2.38)	0.92 (0.77–1.09)	1.17 (0.86–1.58)	1.00 *
***miR-129***	1.75 (0.92–3.32)	1.00 *	1.50 (0.85–2.64)	1.00 *
***miR-935***	2.67 (1.09–6.52)	0.92 (0.77–1.09)	1.33 (0.76–2.35)	1.00 *
***PHACTR3***	1.50 (0.85–2.64)	0.83 (0.65–1.07)	1.50 (0.85–2.64)	0.92 (0.77–1.09)
***PRDM14***	1.33 (0.89–1.99)	1.00 *	1.00 *	1.00 *
***SST***	1.00 *	1.00 *	1.50 (0.95–2.38)	0.83 (0.65–1.07)
***ZIC1***	1.00 *	1.00 *	1.00 *	1.00 *
**Panel *GHSR/MAL***	1.43 (0.95–2.14)	0.92 (0.77–1.09)	1.00 *	1.00 *

* CI not estimable, since there was a perfect match in positive and negative outcomes between both fractions. Abbreviations: CI, confidence interval.

## Supplementary Materials

The following are available online at https://www.mdpi.com/2072-6694/12/4/859/s1, Table S1: Sensitivity and specificity of the single methylation markers and marker panel GHSR/MAL for bladder cancer diagnosis in full void, urine pellet and supernatant, Table S2: Cut-offs for the nine methylation markers.
