# Supplementary material for: Comparative Analysis of Urine Fractions for Optimal Bladder Cancer Detection Using DNA Methylation Markers
Source: Cancers (Basel). 2020 Apr 2;12(4):859. doi: 10.3390/cancers12040859 (PMC7226114; doi:10.3390/cancers12040859)
Supplement: Supplementary file 1 [file cancers-12-00859-s001.pdf]

Table S1. Sensitivity and specificity of the single methylation markers and marker panel GHSR/MAL for bladder cancer diagnosis in full void, urine pellet and supernatant.

Markers	Full void		Pellet		Supernatant	
	Sens (95% CI)	Spec (95% CI)	Sens (95% CI)	Spec (95% CI)	Sens (95% CI)	Spec (95% CI)
**FAM19A4**	15.4 (1.9 – 45.4)	100.0 (73.5 – 100.0)	14.3 (1.8 – 42.8)	100.0 (73.5 – 100.0)	14.3 (1.8 – 42.8)	100.0 (73.5 – 100.0)
**GHSR**	38.5 (13.9 – 68.4)	100.0 (73.5 – 100.0)	64.3 (35.1 – 87.2)	100.0 (73.5 – 100.0)	61.5 (31.6 – 86.1)	100.0 (73.5 – 100.0)
**MAL**	46.2 (19.2 – 74.9)	100.0 (73.5 – 100.0)	64.3 (35.1 – 87.2)	91.7 (61.5 – 99.8)	53.8 (25.1 – 80.8)	100.0 (73.5 – 100.0)
**miR-129**	30.8 (9.1 – 61.4)	100.0 (73.5 – 100.0)	50.0 (23.0 – 77.0)	100.0 (73.5 – 100.0)	46.2 (19.2 – 74.9)	100.0 (73.5 – 100.0)
**miR-935**	23.1 (5.0 – 53.8)	100.0 (73.5 – 100.0)	57.1 (28.9 – 82.3)	91.7 (61.5 – 99.8)	30.8 (9.1 – 61.4)	100.0 (73.5 – 100.0)
**PHACTR3**	30.8 (9.1 – 61.4)	100.0 (73.5 – 100.0)	50.0 (23.0 – 77.0)	83.3 (51.6 – 97.9)	42.9 (17.7 – 71.1)	100.0 (73.5 – 100.0)
**PRDM14**	46.2 (19.2 – 74.9)	100.0 (73.5 – 100.0)	64.3 (35.1 – 87.2)	100.0 (73.5 – 100.0)	50.0 (23.0 – 77.0)	100.0 (73.5 – 100.0)
**SST**	46.2 (19.2 – 74.9)	100.0 (73.5 – 100.0)	42.9 (17.7 – 71.1)	100.0 (73.5 – 100.0)	69.2 (38.6 – 90.9)	83.3 (51.6 – 97.9)
**ZIC1**	53.8 (25.1 – 80.8)	100.0 (73.5 – 100.0)	50.0 (23.0 – 77.0)	100.0 (73.5 – 100.0)	53.8 (25.1 – 80.8)	100.0 (73.5 – 100.0)
**Panel GHSR/MAL**	53.8 (25.1 – 80.8)	100.0 (73.5 – 100.0)	78.6 (49.2 – 95.3)	91.7 (61.5 – 99.8)	66.7 (34.9 – 90.1)	100.0 (73.5 – 100.0)

Abbreviations: CI: Confidence interval.

For full void we used cut-offs from our previous study [6]. For urine pellet and supernatant we determined new cut-offs. All cut-offs were calculated with Youden's J index [22].

Table S2. Cut-offs for the nine methylation markers.

Markers / Urine fraction	FAM19A4	GHSR	MAL	miR-129	miR-935	PHACTR3	PRDM14	SST	ZIC1
**Full void**	0.7450	-1.7287	2.6559	2.2329	-2.8050	-7.1427	1.3946	-2.7370	-2.3310
**Pellet**	1.7934	-2.5449	-3.2495	0.8365	-10.7835	-10.2051	0.3139	-2.9880	-2.9718
**Supernatant**	1.8513	-2.0550	1.9837	-0.5341	-3.3143	-8.8637	2.1462	-4.8401	-3.1959

For full void, cut-offs from our previous study were used [6]. For urine pellet and supernatant, we determined new cut-offs. All cut-offs were calculated with Youden's J index [22].
